# Preparation Prerequisites for Effective Irrigation of Apical Root Canal: A Critical Review

**DOI:** 10.4317/jced.54117

**Published:** 2017-10-01

**Authors:** Dimitrios Tziafas, Dana Alraeesi, Reem Al Hormoodi, Maamoun Ataya, Hessa Fezai, Nausheen Aga

**Affiliations:** 1 DDS, PhD, Professor in Endodontics, Hamdan Bin Mohamed College of Dental Medicine, MBR University of Medicine and Health Sciences, DHCC Dubai, UAE; 2 BDS, Dentist, resident of Postgraduate Master Program in Endodontics, Hamdan Bin Mohamed College of Dental Medicine, MBR University of Medicine and Health Sciences, DHCC Dubai, UAE

## Abstract

**Background:**

It is well recognized that disinfection of the complex root canal system at the apical root canal remains the most critical therapeutic measure to treat apical periodontitis.

**Material and Methods:**

Observational and experimental data in relation to the anatomy of the apical root canal in different tooth types and the cross sectional diameters of the apical part of the most commonly used hand and rotary files are critically reviewed.

**Results:**

The present data analysis confirm that the challenging issue of antibacterial efficacy of modern preparation protocols in non-surgical endodontics requires more attention to apical root canal irrigation as a balance between safety and effectiveness. *Ex vivo* investigations clearly indicate that a specific design of the chemo-mechanical preparation is needed at the onset of RCT, more particularly in infected teeth. Design should be based on specific anatomical parameters, and must determine the appropriate size and taper of preparation as pre-requirements for effective and safe apical irrigation.

**Conclusions:**

The optimal irrigation protocols might be designed on the basis of technical specifications of the preparations procedures, such as the penetration depth, the type of the needle, the required time for continuous irrigant flow, the concentration of NaOCl, and the activation parameters.

** Key words:**Endodontics, root canal treatment, instrumentation, irrigation, apical root canal.

## Introduction

The objective of root canal treatment (RCT) is to prevent or treat apical periodontitis, which is the sequence of microbial colonization in the root canal system. Chemo-mechanical preparation is considered as the most essential procedure of RCT aiming to clean and shape the total root canal system, more especially to eliminate microorganisms and pathologic debris from this complex tooth area. A variety of instruments and techniques in combination with disinfecting irrigation solutions and intracanal medications have been proposed for the chemo-mechanical preparation of infected root canals. The cleaning and shaping efficiency of root canal instruments, aim to achieve a well-tapered root canal form, sufficient for the required irrigant flow in the whole canal and optimal 3D obturation. The objectives of chemomechanical preparation is continuously evaluated in *ex vivo* studies during the last two decades, more particularly after introduction of the rotary files in RCT (1-3).

It is well recognized that the biological prerequisite for treatment of apical periodontits is the elimination of pathogenic bacteria colonizing the apical part of the root canals at subcritical level compatible with periapical tissue healing (4,5). Studies have sho-wed that the current instrumentation and irrigation techniques are not completely effective in the elimination of debris and bacteria from the apical part of the canal (6-8). Apart the narrow and complex apical root canal morphology, the difficulty in the removal of bacterial debris from the apical canal has been attributed to the inadequate flushing of irrigants (6). Variations in the shape and the diameters of the apical root canal space, affects the dynamics of irrigant flow and subsequently the disinfecting and dissolution effects of irrigation (8). Furthermore, the apical root canal irrigation must be considered as a challenging issue of RCT, a balance between effectiveness and safety due to the risks for debris and toxic irrigants extrusion to the periapical tissues (8-10).

The aim of the study is to critically review observational data from relative *ex vivo* investigations in focusing on the minimal prerequisites for efficient and safe apical canal irrigation during the RCT of infected teeth. Data were systematically reviewed as follows:

i. The cross sectional diameter of the apical part of the most commonly used hand and rotary files were calculated according to their technical specifications.

ii. The variation of diameters in the apical root canal in different tooth types was reviewed by bringing together data from classical and recent literature.

iii. The observations from experimental studies approaching the risk for apical irrigant extrusion in relation to the distance of the apex and the size of apical enlargement were set.

## Material and Methods

-Data Sources and Resources Selection 

This review is based on a comprehensive literature search using the Medline/Pubmed data base covering the period from 1960 to early 2016. The database search was performed using the keywords “apical root canal morphology”, “apical root canal preparation”, “apical root canal irrigation”. Eligible for inclusion in this study were scientific articles that were published in the English language, in international peer-reviewed journals with no limitations implemented by country of origin. The relevant papers included the abstracts and full text of clinical trials (original articles) that met the eligibility criteria. Unpublished research and studies that were reported only in abstract form, review articles, letters to the Editor, clinical guidelines and clinical studies and case reports were not considered for inclusion.

Titles and abstracts were screened and then full texts of all potentially relevant publications were obtained and reviewed. Full paper copies of peer-reviewed papers were acquired electronically and cross references were further screened to identify relevant studies. Any disagreements on study inclusion and exclusion criteria were discussed and resolved either by consulting a third reviewer.

-Data interpretation 

Part I. Data including the cross sectional diameter at 5 different file levels (D0-D5) of the most commonly used hand and rotary files according to their technical specifications were calculated by one reviewer. All hand files no #25 - #50 with 3 different tapers (0.2, 0.4, 0.6), the Protaper next and Universal systems (Dentsply Maillefer), and the iRace (FKG Dentaire), Revo, K3 (Micro-Mega, Besançon, France), Wave –one (Dentsply Maillefer) and Reciproc systems (VDW Dental) were comparatively evaluated.

Part II. Data concerning the variation of diameter at 5 different root canal levels (0-5 mm from the apex) in different tooth types were extracted by two independent reviewers and placed in different tables for maxillary and mandibular teeth.

Part III. Data from *ex vivo* investigations on extrusion of irrigants in relation to the methods for apical canal instrumentation and irrigation were extracted by two independent reviewers and placed together in a table.

## Results

-Review

Part I. In  Table 1 the cross sectional diameters of the apical part (levels 0-5 mm/ D0 – D5) for the ten selected systems of hand or rotary files are shown.

**Table 1:**
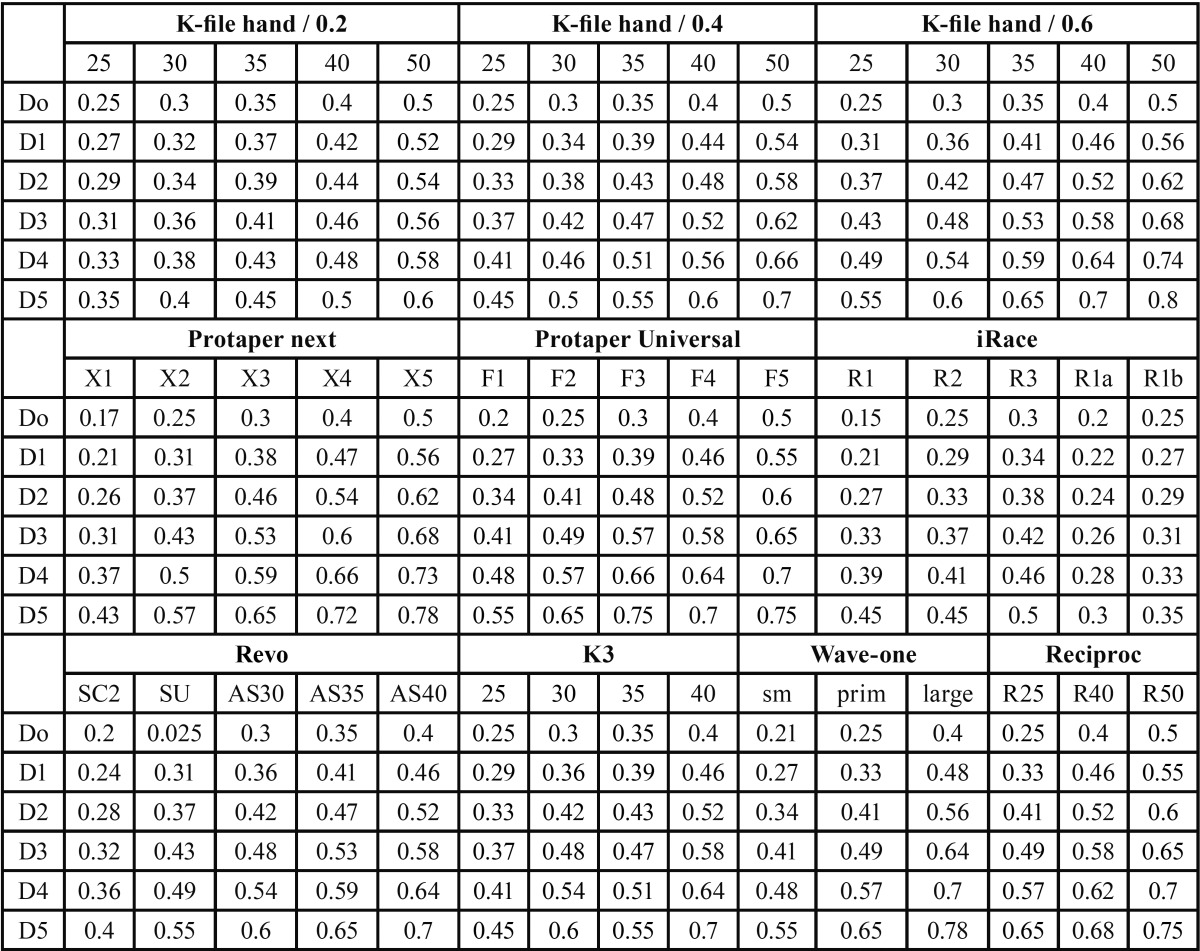
Do –D5 diameters of 10 different systems of hand or rotary files.

Part II. Five and eight studies including data for maxillary and mandibular teeth respectively were included in the final evaluation. In  Table 2 and  Table 3 the studies are shown with their reference data minor and maximum diameters of cross sections of teeth at a distance of 1-5 mm from the apex. The range of diameters, when they were available, were also set.

**Table 2:**
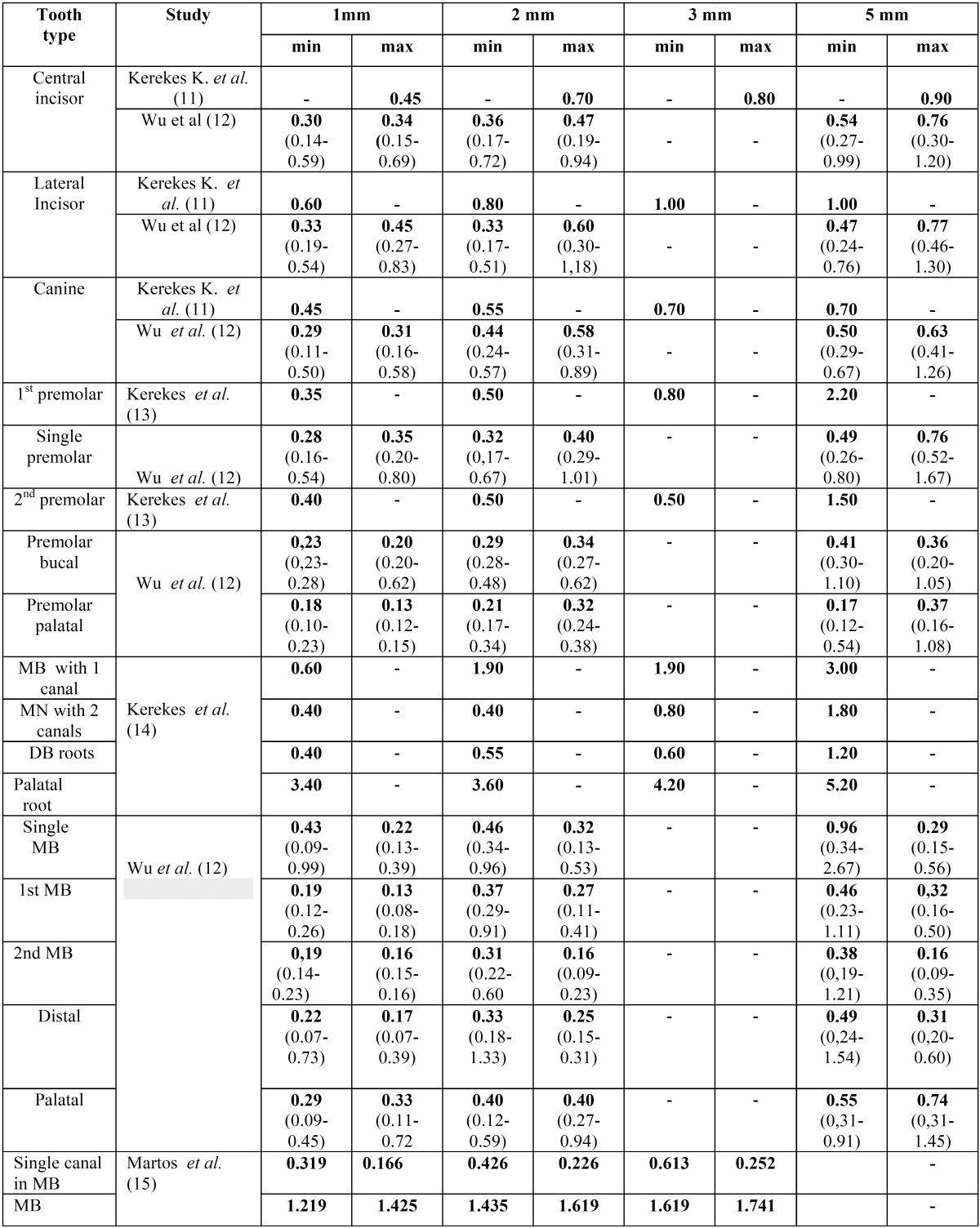
Average minor and maximum diameters of cross sections of maxillary teeth at a distance of 1-5 mm from the apex (range at parentheses).

**Table 3:**
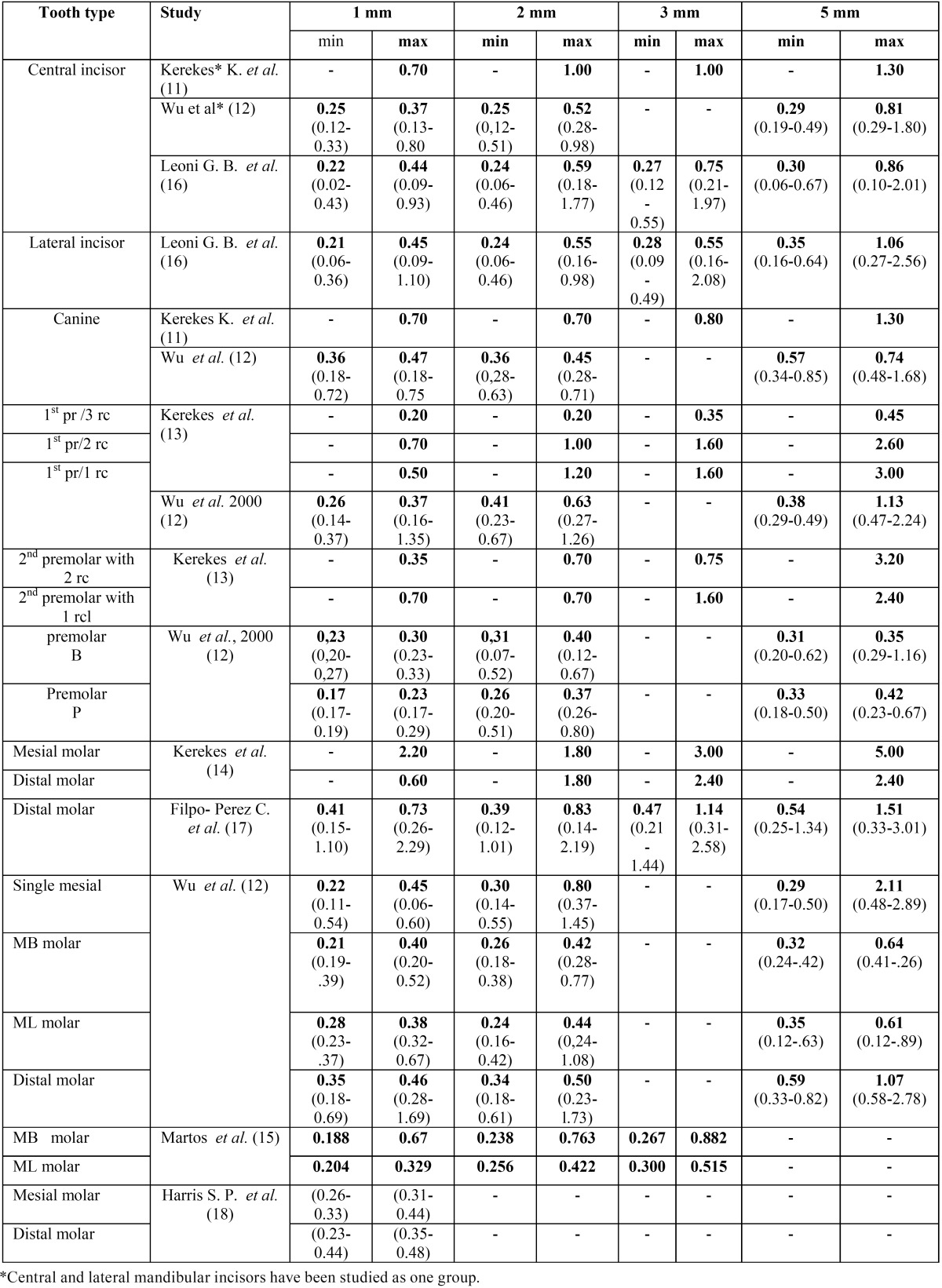
Average minor and maximum diameters of cross sections of mandibular teeth at a distance of 1-5 mm from the apex (range at parentheses).

-Data analysis

**Figure 1:**
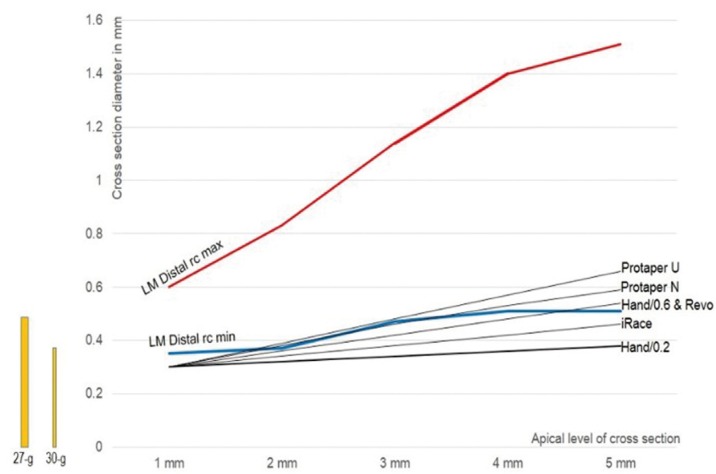
The median value of minimal (blue) and maximum (red) canal diameter in the distal root of the lower molar (data from table III). The corresponding diameters of the hand files with taper 0.2 and 0.6, and rotary files Protaper Universal and Next, iRace and Revo (data from table I). All file systems potentially leave unprepared or almost unprepared root canal space in this type of root canal. The bars in yellow show the minimal diameter that is required for penetration of needles 27-g and 30-g during apical canal irrigation.

**Figure 2:**
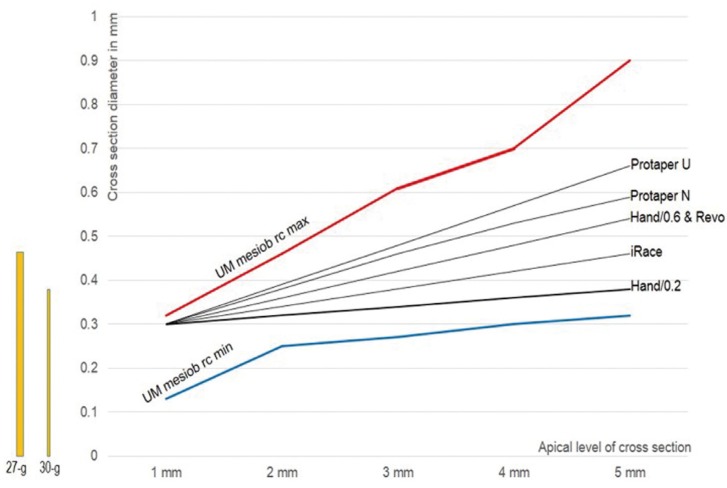
The median value of minimal (blue) and maximum (red) canal diameter in the mesiobuccal 1 root canal of the maxillary molar (data from table II). The corresponding diameters of the hand files with taper 0.2 and 0.6, and rotary files Protaper Universal and Next, iRace and Revo (data from table I). The selected file systems with various tapers seem to potentially leave unprepared root canal space depended in this type of root canal. The bars in yellow show the minimal diameter that is required for penetration of needles 27-g and 30-g during apical canal irrigation.

The comparative data of two root canals (taken as indicative examples) and five file systems were analysed. Median maximum and minimum vales of the apical canal diameters in the distal root canal of mandibular molar and the mesiobuccal-1 root canal of maxillary molars were used. Data are shown in figures 1 and 2. The most characteristic major points that can be raised are the following.

a. In both root canal types all file systems potentially leave unprepared root canal, when the median value of maximum diameters are considered. In these cases the 27-g needle tip easily reaches at a distance of 3 mm from the apex.

b. In the case of distal root canal of mandibular molar some file systems potentially leave unprepared root canal, even when median value of minor diameters are considered. In this case only the 30-g needle tip reaches at a distance of 3 mm from the apex.

c. In the case of mesiobuccal-1 root canal of maxillary molar all file systems seem to prepare adequately the root canal, when the median value of minor diameters are considered. In this case, as well as in the distal root canal of the mandibular molar, the 27-g needle tip cannot reach at a distance less than 4 mm from the apex after their preparation with files systems with reduced taper.

These *ex vivo* observations and data concerning the risk of apical irrigant extrusion as shown in table IV, clearly indicate that issues related to apical canal irrigation should be re-considered. Among these issues the need of minimal size of apical enlargement, the required file taper, the appropriate needle-tip and the safe distance from the apex seem to represent important prerequisites for effective and safe irrigation during RCT of infected teeth.

## Discussion

Irrigation fulfils several important chemical and microbiological functions. According to Haapasalo *et al.* (4) irrigation is the only way to remove tissue remnants and bacteria in planktonic and biofilm forms, from the complex areas of the root canal walls that are not touched by mechanical instrumentation. Literature data concerning diameters of apical root canals in different tooth types, reviewed in this article, show extreme variation in the shape of this important endodontic tissue area. Taking into consideration together these data with the shaping efficiency of different systems/instruments in the today endodontic practice, we can safely accept previous microbiological observations that mechanical preparation cannot achieve predictable disinfection (19,20). The elimination of the remaining bacteria after instrumentation of the root canal remains a challenging issue in terms of the widely recognized biological principles in RCT. It has been reasonably suggested that instrumentation to a large apical size (#50 and more) can remove more infected dentin and more bacterial cells from the root canal. However, the apical enlargement of curved canals to sizes more than #40 with maintenance of original root canal path is not always clinically accessible. Thus, it remains to be clarified what is the optimal disinfection protocol, irrigation and intracanal mediacation, for the instrumented root canals.

The untouched dentinal walls by the files in the root canals, more particularly in the apical part of oval-shaped ones, underline the need for more detailed technical specifications for the effective control of all parameters of irrigation. Furthermore, other features of complex apical root canal anatomy, the lateral canals and isthmuses which cannot be reached by the mechanical canal preparation, emphasizes also the need for understanding of requirements of apical canal irrigation, in terms of dynamics of fluid flow. Deeper penetration of irrigant with increased wall shear stress effect can achieve better results in removal planktonic and biofilm bacteria (21). However, increased shear stress result also in increased pressure of the irrigant at the apex, which provides higher risks for irrigant and /or infected debris extrusion.

*Ex vivo* investigations on the rate of apical extrusion in relation to instrumentation and irrigation parameters of RCT have showed interesting findings with a clinical significance. Altundasar *et al.* (22) performed preparation with Protaper or iRace files in mandibular premolars with single canals. Highest fluid extrusion was seen with ProTaper files and regular needle irrigation. The lowest irrigant extrusion was observed with the iRaCe system combined with a side-vented irrigation needle. Boutsioukis *et al.* (10) performed an *ex vivo* study with straight root canal prepared to size 35, 0.06 taper NaOCl with open-ended and close-ended needles. Manual dynamic, sonic and ultrasonic agitation were compared. Significantly more irrigant extruded with the open- than the closed-ended needles . Extrusion decreased as needles moved away from the apex. The effect of apical constriction diameter was not significant. More extrusion with manual dynamic agitation was found. Yost *et al.* (23) prepared mandibular and maxillary central incisors to size 35/.04 and 55/.04. Endo vac vs side vended needles were examined for extrusions of 6% NaOCl. They reported extrusion 40% after irrigation with the needle and ultrasonic activation, while extrusion only 10% after the use of EndoVac.

The present data indicate that for optimal therapeutic results, a specific design of the chemomechanical preparation is needed at the onset of RCT in infected teeth. Anatomical parameters, such as the type of the tooth, the shape of the root canal, the existing curvature, etc must determine the appropriate size and taper of preparation of the apical root canal, which is required for the optimal antibacterial efficacy of apical irrigation. The optimal irrigation protocols might be further specified by the technical specifications of the irrigation procedure, such as the penetration depth of the needle, the time of continuous irrigant flow, its concentration, activation parameters etc. in order to provide the best apical canal walls cleaning and effective and safe apical root canal irrigation (4,24).

In conclusion these data strongly confirm the previously stated assumptions that the apical root canal, which is particularly important for the successful outcome of RCT in infected teeth, poses a special challenge to irrigation as the balance between safety and effectiveness. Further critical analysis of observational data will lead scientists to design the directions of new protocols for effective and safe instrumentation and irrigation in the clinically important area of apical root canal.
